# Landmark survival as an end-point for trials in critically ill patients – comparison of alternative durations of follow-up: an exploratory analysis

**DOI:** 10.1186/cc7988

**Published:** 2009-08-04

**Authors:** Gopal Taori, Kwok M Ho, Carol George, Rinaldo Bellomo, Steven AR Webb, Graeme K Hart, Michael J Bailey

**Affiliations:** 1 Department of Intensive care, Austin Hospital, Studley Road, Melbourne 3084, Australia; 2 Department of Intensive Care, Royal Perth Hospital, Wellington Street, Perth 6001 Australia; 3 Clinical Associate Professor, School of Population Health, University of Western Australia, Stirling Highway, Crawley 6009, Australia; 4 ANZICS CORE Group, Australian and New Zealand Intensive Care Society, 10 Ievers St, Carlton 3053, Australia; 5 ANZIC-RC, School Public Health & Preventive Medicine, Monash University Alfred Hospital, Commercial Road, Melbourne 3181, Australia

## Abstract

**Introduction:**

Interventional ICU trials have followed up patients for variable duration. However, the optimal duration of follow-up for the determination of mortality endpoint in such trials is uncertain. We aimed to determine the most logical and practical mortality end-point in clinical trials of critically ill patients.

**Methods:**

We performed a retrospective analysis of prospectively collected data involving 369 patients with one of the three specific diagnoses (i) Sepsis (ii) Community acquired pneumonia (iii) Non operative trauma admitted to the Royal Perth Hospital ICU, a large teaching hospital in Western Australia (WA cohort). Their in-hospital and post discharge survival outcome was assessed by linkage to the WA Death Registry. A validation cohort involving 4609 patients admitted during same time period with identical diagnoses from 55 ICUs across Australia (CORE cohort) was used to compare the patient characteristics and in-hospital survival to look at the Australia-wide applicability of the long term survival data from the WA cohort.

**Results:**

The long term outcome data of the WA cohort indicate that mortality reached a plateau at 90 days after ICU admission particularly for sepsis and pneumonia. Mortality after hospital discharge before 90 days was not uncommon in these two groups. Severity of acute illness as measured by the total number of organ failures or acute physiology score was the main predictor of 90-day mortality. The adjusted in-hospital survival for the WA cohort was not significantly different from that of the CORE cohort in all three diagnostic groups; sepsis (*P *= 0.19), community acquired pneumonia (*P *= 0.86), non-operative trauma (*P *= 0.47).

**Conclusions:**

A minimum of 90 days follow-up is necessary to fully capture the mortality effect of sepsis and community acquired pneumonia. A shorter period of follow-up time may be sufficient for non-operative trauma.

## Introduction

Mortality is the most clinically relevant and commonly used primary outcome measure for phase III trials in intensive care. However, the optimal duration of follow-up for the determination of mortality in such trials is uncertain [1,2]. Interventional ICU trials have followed up patients for different durations [3-7]. Furthermore, some trials have censored follow up at time of hospital discharge ignoring any subsequent out-of-hospital deaths [8,9]. Such variability creates confusion, leads to controversy and makes meta-analyses of trials with different times of mortality assessment difficult to interpret. Measurement of mortality at 28-days or censoring at hospital discharge have logistic advantages but as many as one-third of critically ill patients may still be in hospital after 28 days and deaths can still occur soon after hospital discharge [3]. Longer follow up time, however, may make it difficult to distinguish between the effects of critical illness (or the studied interventions) from those of underlying age and co-morbidities [10]. Follow up for longer time periods, especially where this extends beyond hospital discharge, is more difficult and costly. The ideal period of follow up would be up to a time point by which the effects of critical illness remain powerful independent determinants of outcome and before pre-existing factors, such as age and co-morbidity, can have a marked and confounding impact on survival [11].

The Australian and New Zealand Intensive Care Society (ANZICS) Centre for Outcome and Resource Evaluation (CORE) Adult Patient Database (APD) gathers information about the vast majority of admissions of critically ill patients from various intensive care units (ICUs) across Australia and New Zealand but currently does not follow up patients beyond hospital discharge [12]. However, in an embedded cohort of ICU patients treated at the Royal Perth Hospital, which is a large university teaching hospital in Western Australia (WA cohort), such information is available [11]. Western Australia is geographically isolated and has a low rate of emigration [11] and, as such, loss to medium-term and long-term survival follow-up by the Western Australian Death Registry is very low [13].

We hypothesized that, if the characteristics and short-term outcomes of patients in the WA cohort and the various ICUs from Australia (as identified within the two databases) were comparable, then the follow-up data of the patients in WA cohort could be used to estimate the likely in-hospital and out-of-hospital long-term survival of critically ill patients in Australia.

## Materials and methods

We conducted a retrospective analysis of prospectively collected data from two large, related databases. Access to the data was granted by the ANZICS CORE Management Committee in accordance with standing protocols. Data are collected primarily for ICU Outcome Peer Review under Quality Assurance Legislation of the Commonwealth of Australia (Part VC Health Insurance Act 1973, Commonwealth of Australia). Such data are collected and transferred from hospitals to the database with government support and funding. Hospital data are submitted by or on behalf of the ICU Director and results are reported back to the Director. Each hospital allows subsequent data use as appropriate under the ANZICS CORE standing procedures and in compliance with the ANZICS CORE Terms of Reference [14] and waives the need for informed consent. CORE does not hold individual patient identifying data and as such informed consent has been waived and specific ethical approval was not required. Hospital identifying data is held encrypted in the CORE database and was not released for this study. The WA linked data had the patient name and address removed and the Western Australian Confidentiality of Health Information Committee approved the study.

The study cohort consisted of all patients over 18 years of age who were admitted to ICU from emergency departments between 1 January, 2001 and 31 December, 2002 with one of three acute physiology and chronic health evaluation score (APACHE) II diagnoses [15]: sepsis of any etiology; community acquired pneumonia or non-operative trauma.

The data for the WA cohort were extracted from the Royal Perth Hospital ICU database. In this study, the survival outcome after hospital discharge of the WA cohort was assessed on 31 December 2003 by linkage to the WA death registry [11,16]. The APACHE III-related physiology, diagnostic and chronic health data of admissions from 55 Australian ICUs were extracted from the ANZICS CORE adult patient database (CORE cohort). In the CORE cohort, only ICUs that consistently contributed data over a longer period (2001 to 2006) were included, because the quality of the data from these contributing sites was likely to be more consistent than from units that were discontinuous contributors. Sites with missing data for two or more years were also excluded. These CORE cohort APACHE III data were converted to APACHE II data using a specific algorithm [17,18].

The in-hospital and subsequent survival data of the WA cohort at different time points after ICU admission was used to assess whether a 'plateau' was observed. These data were then further analyzed to determine the incidence of death after hospital discharge and the quantum effect of various variables on survival at different time points. A formal landmark survival analysis was performed with the landmark time point chosen as ICU discharge. The variables assessed included age, gender, Charlson co-morbidity index [19], Acute physiology score component of the APACHE II score [15], and maximum number of organ failure during ICU admission. The definition of organ failure used for the study has been described previously [11,20]. In the assessment of non-operative trauma, Glasgow Coma Score (GCS) was also analyzed in addition to other variables. The data analyzed had the patient name and address removed and the study was approved by the Royal Perth Hospital Ethics Committee and the Western Australian Confidentiality of Health Information Committee, which waived the need for informed consent. The in-hospital survival of the WA cohort was then compared with the CORE cohort to look at the applicability of its long-term follow-up data to a larger population.

### Statistical analysis

Continuous data with a near normal distribution are presented as mean and standard deviation and data with a skewed distribution were expressed as median and interquartile range. Categorical variables and data with a skewed distribution are analysed by chi-squared and Mann-Whitney test, respectively. Kaplan-Meier survival analysis and log-rank test was used to compare the difference in hospital survival between the WA cohort and ANZICS APD cohort. Single variable and multivariable analyses were performed using logistic regression for binomial outcomes and reported using odds ratios (95% confidence interval (CI)) and Cox proportional hazard regression for time to death with results reported using hazard ratios (95% CI). Survival analysis was performed with survival time measured from both ICU admission and ICU discharge. Multivariable models were constructed using both stepwise selection and backward elimination procedures before undergoing a final assessment for clinical and biological plausibility. Statistical analysis was performed using SAS version 9.1 (SAS Institute, Cary, NC, USA) and SPSS statistical software (version 13.0 for Windows, SPSS Inc., Chicago, IL, USA). A two-sided *P *value of 0.05 was considered to be statistically significant.

## Results

**Figure 1:**
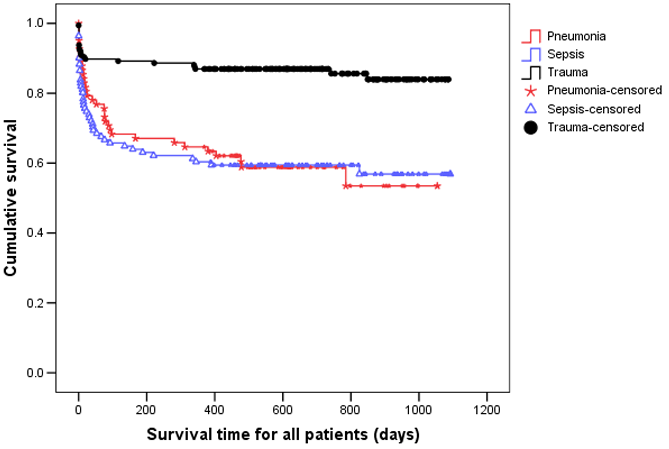
Kaplan Meier curves for time to death from intensive care unit admission for the three types of diagnosis. Survival time is expressed in days.

**Figure 2:**
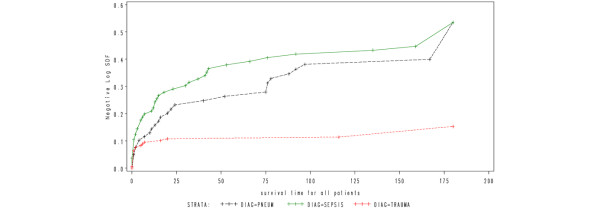
Cumulative hazard function for time to death from intensive care unit admission for the three types of diagnosis.  Note, for increased interpretability, all survival times greater than 180 days have been truncated to 180 days.

**Figure 3:**
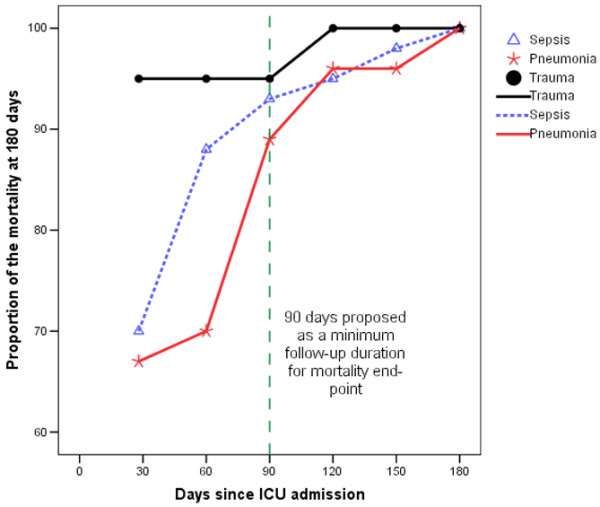
Mortality at different time point as a proportion of cumulative mortality at 180 days after ICU admission.  ICU = intensive care unit.

**Figure 4:**
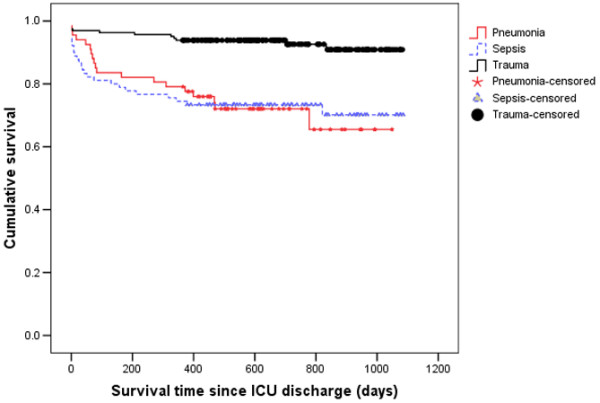
Kaplan Meier curves for time to death from ICU discharge for the three types of diagnosis.  Survival time is expressed in days. ICU = intensive care unit.

**Figure 5:**
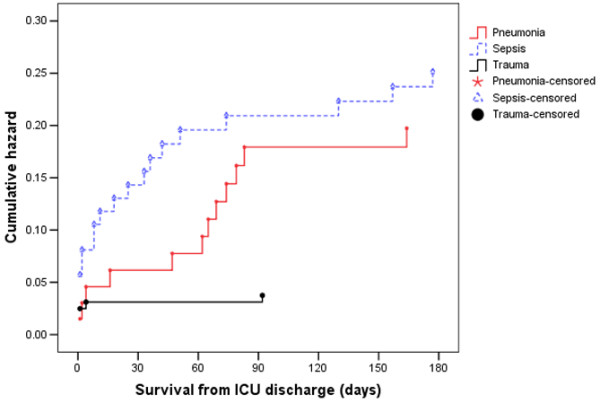
Cumulative hazard function for time to death from ICU discharge for the three types of diagnosis.  Note, for increased interpretability, all survival times greater than 180 days have been truncated to 180 days. ICU = intensive care unit.

When considering patients with pneumonia or sepsis, 28-day mortality only effectively captured 67% and 70% of deaths that occurred within six months of ICU admission. By considering 90-day mortality, the proportion of deaths captured increased to 89% and 93%, respectively (Figures 1 and 2). The absolute increase in mortality between 90 and 180 days in these two diagnostic subgroups was relatively small (2.7%, 95% CI = 15% to 9.7%; and 3.6%, 95% CI = 17.5% to 10.4%, respectively; Figure 3 and Table 1). As for the patients with non-operative trauma, mortality rate appeared to 'plateau' well before 28 days (Figure 2). These results remained consistent when considering post ICU survival (Figures 4 and 5).

**Table 1:** Mortality at different time points and the percentage of deaths that occur within 180 days captured at each time point

Time period (days)	Sepsis (n = 111)	Pneumonia (n = 82)	Trauma (n = 176)
	Cumulative total number of deaths (% of deaths captured)	Cumulative total number of deaths (% of deaths captured)	Cumulative total number of deaths (% of deaths captured)

28	28 (70)	18 (67)	18 (95)

60	35 (88)	19 (70)	18 (95)

90	37 (93)	24 (89)	18 (95)

120	38 (95)	26 (96)	19 (100)

150	39 (98)	26 (96)	19 (100)

180	40 (100)	27 (100)	19 (100)

Single-variable analysis showed the APACHE score to be the most consistent predictor of mortality but not a statistically significant predictor of time to death after ICU discharge for either pneumonia or sepsis (Table 2). GCS was a consistent predictor of survival for trauma-related mortality, while patient age was a consistent predictor for mortality in the pneumonia subgroup.

**Table 2:** Single variable and multivariable analysis for prediction of death and survival (*P < 0.05)

Single variable analysis							
**Diagnosis**	**Variables**	**28 day**	**90 day**	**180 day**	**All mortality**	**Time** **to death (TTD)**	**TTD from** **ICU discharge**

Pneumonia	Age	1.08 (1.03–1.13)*	1.09 (1.04–1.14)*	1.08 (1.04–1.13)*	1.07 (1.03–1.10)*	1.06 (1.03–1.09)*	1.05 (1.01–1.09)*

	APACHE score	1.11 (1.02–1.21)*	1.08 (1.01–1.17)*	1.09 (1.02–1.18)*	1.07 (1.00–1.15)*	1.06 (1.01–1.11)*	1.04 (0.97–1.11)
	
	Charlson	1.07 (0.88–1.29)	1.12 (0.94–1.33)	1.13 (0.95–1.35)	1.11 (0.93–1.33)	1.06 (0.96–1.18)	1.09 (0.96–1.22)
	
	GCS	1.30 (0.82–2.05)	1.01 (0.81–1.26)	1.00 (0.81–1.23)	1.00 (0.82–1.23)	1.01 (0.87–1.19)	0.93 (0.80–1.09)
	
	Organ score	1.44 (1.05–1.97)*	1.39 (1.04–1.84)*	1.56 (1.16–2.11)*	1.41 (1.07–1.86)*	1.30 (1.08–1.57)*	1.25 (0.96–1.62)
	
	Male	0.68 (0.22–2.05)	0.74 (0.28–1.96)	1.11 (0.44–2.82)	1.11 (0.46–2.68)	1.03 (0.52–2.06)	1.19 (0.47–2.99)

Sepsis	Age	1.01 (0.99–1.04)	1.02 (1.00–1.05)	1.02 (1.00–1.05)	1.03 (1.01–1.06)*	1.02 (1.00–1.04)*	1.05 (1.02–1.08)*

	APACHE score	1.07 (1.01–1.13)*	1.07 (1.02–1.13)*	1.06 (1.00–1.11)*	1.05 (1.00–1.11)	1.05 (1.01–1.09)*	1.04 (0.99–1.10)
	
	Charlson	1.04 (0.87–1.23)	1.00 (0.85–1.18)	1.07 (0.91–1.25)	1.15 (0.98–1.35)	1.08 (0.98–1.20)	1.15 (1.02–1.30)*
	
	GCS	0.94 (0.84–1.04)	0.89 (0.8–0.99)*	0.9 (0.81–1.01)	0.9 (0.81–1.00)	0.93 (0.87–1.00)*	0.92 (0.84–1.00)
	
	Organ score	1.67 (1.26–2.23)*	1.42 (1.12–1.8)*	1.38 (1.09–1.74)*	1.38 (1.10–1.73)*	1.28 (1.09–1.50)*	1.14 (0.92–1.41)
	
	Male	0.77 (0.33–1.81)	1.00 (0.45–2.2)	1.07 (0.49–2.33)	1.06 (0.5–2.25)	1 (0.56–1.81)	1.03 (0.46–2.30)

Trauma	Age	1.00 (0.97–1.03)	1.00 (0.97–1.03)	1.01 (0.98–1.04)	1.01 (0.99–1.04)	1.01 (0.99–1.03)	1.03 (1.00–1.07)

	APACHE score	1.28 (1.16–1.41)*	1.28 (1.16–1.41)*	1.25 (1.15–1.36)*	1.22 (1.13–1.31)*	1.18 (1.12–1.24)*	1.13 (1.05–1.22)*
	
	Charlson	0.87 (0.32–2.35)	0.87 (0.32–2.35)	0.85 (0.31–2.32)	0.72 (0.24–2.13)	0.74 (0.25–2.18)	0 (0–.)
	
	GCS	0.62 (0.49–0.79)*	0.62 (0.49–0.79)*	0.70 (0.59–0.83)*	0.77 (0.69–0.87)*	0.78 (0.70–0.88)*	0.87 (0.76–0.99)*
	
	Organ score	1.75 (1.30–2.36)*	1.75 (1.30–2.36)*	1.73 (1.29–2.31)*	1.66 (1.28–2.16)*	1.46 (1.22–1.75)*	1.44 (1.10–1.87)*
	
	Male	0.68 (0.19–2.47)	0.68 (0.19–2.47)	0.63 (0.17–2.28)	0.44 (0.12–1.54)	0.47 (0.14–1.62)	0.69 (0.15–3.25)

**Multivariable analysis**							

**Diagnosis**	**Variables**	**28 day**	**90 day**	**180 day**	**All mortality**	**Time** **to death (TTD)**	**TTD from** **ICU discharge**

Pneumonia	Age	1.08 (1.03–1.13)*	1.09 (1.04–1.14)*	1.08 (1.03–1.13)*	1.07 (1.03–1.10)*	1.05 (1.02–1.09)*	1.05 (1.01–1.09)*
	
	Organ score			1.54 (1.11–2.15)*		1.26 (1.03–1.55)*	

Sepsis	Age	1.04 (1.01–1.07)*	1.03 (1.00–1.05)*		1.04 (1.01–1.07)*	1.03 (1.01–1.05)*	1.05 (1.02–1.08)*
	
	Organ score	1.45 (1.13–1.85)*	1.47 (1.14–1.89)*	1.38 (1.09–1.74)*	1.45 (1.13–1.85)*	1.31 (1.11–1.54)*	

Trauma	APACHE score	1.28 (1.16–1.41)*	1.28 (1.16–1.41)*	1.25 (1.15–1.36)*	1.22 (1.13–1.31)*	1.18 (1.12–1.24)*	1.13 (1.05–1.22)*

APACHE = Acute Physiology and Chronic Health Evaluation; GCS = Glasgow Coma Scale.

Multivariable analysis showed that markers of acute illness, such as the number of organ failure and APACHE score, were the strongest predictors of mortality for sepsis, community acquired pneumonia and non-operative trauma (Table 2). Although age was also important in patients with community acquired pneumonia and sepsis, co-morbidities did not appear to have an independent predictive value across the three diagnostic subgroups (Table 2).

When the two cohorts were compared patients from the WA cohort were slightly younger, had less co-morbidity, and a longer length of ICU and hospital stay across all three diagnostic subgroups (Table 3). However, their APACHE II predicted mortality and hospital mortality were not statistically significantly different across the three diagnostic subgroups.

**Table 3:** Comparison of the WA and CORE cohorts

	Sepsis			Pneumonia			Trauma		
**Variable**	**WA** **(n = 111)**	**CORE** **(n = 1429)**	***P***	**WA** **(n = 82)**	**CORE** **(n = 1066)**	***P***	**WA** **(n = 176)**	**CORE** **(n = 2114)**	***P***

Age, years (SD)	54.6 (16.9)	60.1 (17.9)	0.001	56.1 (15.7)	61.1 (17.8)	0.003	35.9 (16.27)	42.6 (19.3)	0.001

Male, number (%)	54 (48.6)	792 (55.4)	0.20	47 (57.3)	588 (55.2)	0.73	137 (77.8)	1599(75.6)	0.58

Median APACHE II score (IQR)	22.0 (11.0)	21.0 (13.7)	0.90	20 (9.3)	19 (10)	0.80	13.0 (9.8)	11.0 (10.0)	0.001

Median APACHE II predicted mortality, % (IQR)	45.2 (37.6)	41.6 (43.6)	0.78	35.5 (28.3)	32.2 (31.0)	0.80	6.3 (12.1)	6.2 (12.4)	0.12

Chronic respiratory disease, number (%)	2 (1.8)	126 (8.8)	0.006	8 (9.8)	206 (19.3)	0.04	1 (0.6)	58 (2.7)	0.08

Chronic cardiovascular disease, number (%)	1 (0.9)	140 (9.8)	0.001	1 (1.2)	93 (8.7)	0.01	0 (0)	43 (2.0)	0.07

Chronic renal disease, number (%)	3 (2.7)	105 (7.3)	0.08	3 (3.7)	27 (2.5)	0.47	0 (0)	3 (0.1)	1.00

Chronic liver disease, number (%)	0 (0)	59 (4.1)	0.02	0 (0)	25 (2.3)	0.25	0 (0)	12 (0.6)	0.62

Immunosuppressed state, number (%)	7 (6.3)	185 (12.9)	0.05	5 (6.1)	101 (9.4)	0.43	0 (0)	38 (1.8)	0.11

Median length of ICU stay, days (IQR)	5.1 (7.0)	2.4 (4.9)	0.001	7 (8.3)	3.61 (6.6)	0.001	4.0 (9.8)	2.0(4.7)	0.001

Median length of hospital stay, days (IQR)	18.0 (24.0)	9.9 (16.1)	0.001	15 (11.8)	11.4 (13.5)	0.01	18.0 (26.8)	8.0 (16.7)	0.001

ICU mortality, number (%)*	24 (21.6)	319 (23.0)	0.82	13 (15.9)	169 (16.2)	1.00	17 (9.7)	163 (8.0)	0.47

28-day mortality, number (%)	28 (23.4)	355 (27.9)	0.58	18 (22.0)	190 (20.2)	0.67	18 (10.2)	195 (9.7)	0.79

Hospital mortality, number (%)*	35 (31.5)	417 (30.7)	0.83	20 (24.4)	230 (23.0)	0.79	20 (11.4)	210 (10.5)	0.70

# *P *values were generated by either Mann-Whitney or chi-squared test.

* Intensive care unit (ICU) and hospital mortality outcome of CORE cohort was available only in 2031 and 2010 cases, respectively.

APACHE = Acute Physiology and Chronic Health Evaluation; IQR = interquartile range; SD = standard deviation.

## Discussion

Using the WA data, we found that the mortality of sepsis and community acquired pneumonia reached a plateau by 90 days and that mortality after hospital discharge was common. We further found that at 90 days after ICU admission the severity of acute illness on ICU admission was still the most important predictor of mortality.

We compared the characteristics, severity of illness and in-hospital outcomes of 55 ICUs across Australia (CORE cohort) with those of a cohort of patients with identical diagnoses from a university teaching hospital in Western Australia (WA cohort) for whom long-term outcome was available. We found that the APACHE II-predicted mortality, hospital mortality, and in-hospital survival curves were similar between the WA and CORE cohorts.

This study uses very high quality prospectively collected data (ANZICS CORE APD) that is representative of the ICU patient population in Australia and provides a valid comparator with which to evaluate how general the WA data is [11,12,20]. While acknowledging that there are some differences in the baseline characteristics between the two cohorts, we note that all measures of acute illness severity (the most important predictors of outcome) were statistically equivalent and that the possibility of similar in-hospital survival curves occurring by chance is very low. Therefore, we believe that the long-term survival data of the WA cohort may be reasonably representative of other Australian ICU populations. The ICU practices and post acute hospital care across Australia are similar. Australian ICU practice and outcomes are sufficiently similar to those across the developed world to suggest that studies comparing survival at different landmarks in Australia are likely to have a relevance to practices elsewhere in the developed world.

Many interventional ICU trials have used different durations of follow up with which to assess mortality but the most appropriate duration of follow up is uncertain [3-7]. Our results show that the mortality of sepsis and community acquired pneumonia does not reach a plateau until 90 days after ICU admission and that a substantial proportion of late deaths occur after hospital discharge. Accordingly, assessment of mortality at day 90 and without censoring at hospital discharge is the strategy that is most strongly supported by this analysis. Prolongation of follow up, to 180 days, adds little value. In contrast, duration of follow up to 28 days may well be adequate for patients with ICU admissions due to non-operative trauma.

Epidemiological data shows that severity of illness and organ failure that requires intervention can have a mortality effect long after hospital discharge [21-23] It is thus possible that characteristics of the disease, patient, and interventions in ICU may have a long-term effect on outcomes of ICU patients. In our study multivariable analysis showed that markers of acute illness, such as the number of organ failure and APACHE score, were the strongest predictors of mortality for sepsis, community acquired pneumonia, and non-operative trauma. On the other hand among non-modifiable characteristics only age was important in patients with community acquired pneumonia and sepsis, while co-morbidities did not appear to have an independent predictive value across the three diagnostic subgroups. Although it may be argued that death is not the only patient-centred outcome, it is however one of the most important outcomes studied in many clinical trials. Death, especially long-term survival rate, is often used in many clinical trials as the primary end-point, not only in ICU medicine but also in cardiology and oncology.

This study has several strengths. It formally addresses the important issue of what might be an appropriate duration of follow-up for the assessment of mortality as an outcome. It used high-quality databases for this assessment and confirmed the biological and clinical appropriateness of 90-day follow up by showing that 90 days after ICU admission, the degree of illness severity at ICU admission remained an important predictor of outcome. However, our study also has limitations. Although the WA cohort was comparable with a wider Australian ICU sample in severity of illness and hospital survival, it is still possible that the survival pattern of the two cohorts could be different and we failed to detect such a difference. This seems unlikely given the striking similarity in illness severity, short-term outcome similarities, and the general uniformity of the urban Australian population. It further seems unlikely given that the observations are internally consistent for three separate conditions. However, our results may not be generally applicable to ICU patients in other countries because hospital and healthcare systems vary. Thus, similar studies in other countries are now desirable.

The sample size of the WA cohort in this study was relatively small and the results, therefore, have wide confidence intervals. We acknowledge that our study may not have enough power to truly assess the importance of the selected predictors of mortality. Accordingly, studies involving larger samples may also be desirable to confirm these findings. In addition, we only examined three specific subgroups of critically ill patients. The survival pattern during the first 180 days after the onset of other critical illness may be different in other diagnostic groups [24]. However, these patients have been the subject of many of the randomized controlled trials conducted in ICUs over the past decade and as such, the correct choice of an appropriate landmark survival end point seems particularly important.

## Conclusions

A minimum follow-up time of 90 days without censoring at hospital discharge is necessary to fully capture the mortality effect of community acquired pneumonia and sepsis. For non-operative trauma, a shorter follow-up time appears to be sufficient. This information is important in providing an evidence-based approach in designing and interpreting randomized controlled trials involving these conditions.

## Key messages

• Hospital or 28-day mortality is not an adequate follow up end-point for interventional trials in ICU that involve sepsis and community acquired pneumonia.

• Mortality after hospital discharge is significant up to 90 days when it appears to reach a plateau.

• Severity of illness is the main determinant of mortality at 90 days and, as such, any interventions that aim to attenuate physiological derangement from sepsis or community acquired pneumonia are likely to have a significant effect on mortality up to 90 days.

## Abbreviations

ANZICS: Australian and New Zealand Intensive Care Society; APACHE: Acute Physiology and Chronic Health Evaluation; APD: Adult Patient Database; CI: confidence interval; CORE: Centre for Outcome and Resource Evaluation; GCS: Glasgow Coma Score; ICU: intensive care unit.

## Competing interests

The authors declare that they have no competing interests.

## Authors' contributions

GT designed the study, collected the data, performed the statistical analysis and drafted the manuscript. KMH performed data analysis and helped to draft the manuscript. CG, RB, GH, and SW participated in its design and analysis of the study, and coordinated the drafting of the manuscript. MB performed additional statistical analysis and responded to reviewers. All authors read and approved the final manuscript.
